# Nanomaterials based on hollow gold nanospheres for cancer therapy

**DOI:** 10.1093/rb/rbae126

**Published:** 2024-10-24

**Authors:** You Li, Jing Wang, Ying Li, Ziqiang Luo, Tao Peng, Tao Zou

**Affiliations:** State Key Laboratory of Refractories and Metallurgy, Key Laboratory of Coal Conversion & New Carbon Materials of Hubei Province, School of Chemistry and Chemical Engineering, Wuhan University of Science and Technology, Wuhan 430081, P.R. China; Laboratory for Genetic Engineering of Antibodies and Functional Proteins, Beijing Institute of Pharmacology and Toxicology, Beijing 100850, P.R. China; State Key Laboratory of Refractories and Metallurgy, Key Laboratory of Coal Conversion & New Carbon Materials of Hubei Province, School of Chemistry and Chemical Engineering, Wuhan University of Science and Technology, Wuhan 430081, P.R. China; State Key Laboratory of Refractories and Metallurgy, Key Laboratory of Coal Conversion & New Carbon Materials of Hubei Province, School of Chemistry and Chemical Engineering, Wuhan University of Science and Technology, Wuhan 430081, P.R. China; GEM (Wuhan) Urban Mining Industrial Group Co., Ltd, Wuhan 430415, P.R. China; State Key Laboratory of Refractories and Metallurgy, Key Laboratory of Coal Conversion & New Carbon Materials of Hubei Province, School of Chemistry and Chemical Engineering, Wuhan University of Science and Technology, Wuhan 430081, P.R. China

**Keywords:** Au nanoshell, tumor treatment, photothermal, bioimaging, multimodal therapy

## Abstract

Gold nanoparticles have recently been exploited as versatile nanocarriers in diagnostic and therapeutic drug delivery for cancer nanomedicine, owing to their biocompatibility, low biotoxicity, surface modifiability and plasma optical properties. A variety of gold nanoparticles have emerged for drug delivery, mainly including gold nanorods, gold nanocages, gold nanostars, gold solid nanospheres and hollow gold nanospheres (HGNs). Among these, HGNs have widely been studied for their higher photothermal conversion efficiency, wider spectral absorption range and stronger surface-enhanced Raman scattering compared with solid gold nanospheres. Therefore, nowadays, researchers prefer to use HGNs to other metal nanocarriers, which can not only play the role of controlled-release drugs but also act as photothermal agents for tumor therapy and diagnosis, due to their properties of surface modification. Combined with the Au–S bond on the surface of HGNs, the targeted preparation is loaded to achieve precise drug delivery. With the assistance of the photothermal characteristics of HGNs themselves, the efficacy of loaded drugs in HGNs is enhanced. In addition, HGNs also have vital values in the field of bioimaging, which serve as photothermal imaging agents and Raman scattering-guided preparations due to their surface-enhanced Raman scattering properties to assist researchers in achieving the purpose of tumor diagnosis. In this review, we summarize the synthesis methods of HGNs and the recent application of HGNs-based nanomaterials in the field of cancer diagnosis and therapy. In addition, the issues to be addressed were pointed out for a bright prospect of HGNs-based nanomaterials.

## Graphical abstract

**Figure:**
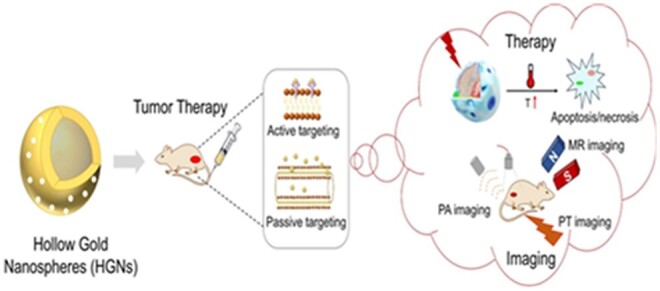

## Introduction

In recent years, research into nanomaterials for cancer therapy has attracted much attention [1–6]. Metal nanoparticles have attracted a lot of interest, especially those with a broad spectral absorption range from ultraviolet to near-infrared (NIR) light [7–9]. Among these, gold nanoparticles stand out as prime candidates for cancer therapy [10–12]. Due to their biocompatibility, low biotoxicity, surface modifiability and plasma optical properties, gold nanoparticles have been widely utilized in cancer diagnosis and treatment [13–16]. Gold nanoparticles exhibit diverse geometries, including nanorods [17, 18], nanocages [19], nanostars [20, 21], solid nanospheres [22], hollow nanospheres [23], etc. These geometries influence the optical properties of gold nanoparticles [24–27]. It has been reported that the photothermal conversion efficiency in hollow structures is significantly higher than that in solid structures. In addition, hollow gold nanospheres (HGNs) exhibit a wider spectral absorption range [28] and stronger surface-enhanced Raman scattering [29–31] than solid gold nanospheres. Moreover, HGNs not only play the role of nanocarriers for controlled drug release [32, 33] but also serve as photothermal agents for cancer therapy.

As the cancer medicine landscape progresses, the diagnosis and treatment of cancer have emerged as pivotal concerns in the realm of material and medical science [34–39]. As a promising treatment method, photothermal therapy (PTT) kills cancer cells by harnessing the local heat generated by organic or noble metal nanomaterials upon laser irradiation [40, 41]. Compared with traditional subcutaneous resection and chemotherapy, PTT shows a lower recurrence rate, less side effects and lower drug resistance [42–46]. HGNs, following laser irradiation, are capable of converting visible and NIR light into thermal energy via surface plasmonic resonance (SPR) after laser irradiation [47–49]. The highly tunable SPR further makes HGNs ideal nanomaterials in photothermal ablation (PTA) as well [50–52]. In addition, the HGNs with outstanding photothermal properties [53, 54] have been considered as excellent candidates for delivering anticancer drugs or fluorescent dyes, thus enabling multimodal therapy [55–69] in tandem with surface modification and hollow structure design.

In order to better play the role of HGNs in cancer diagnosis and treatment, finding a promising method for the synthesis of HGNs is also a top priority for scholars. In recent years, numerous preparation methods for HGNs have been devised, such as the silver nanoparticle sacrificial templates method [70, 71], the cobalt nanoparticle sacrificial templates method [72, 73], the cobalt boride nanoparticle sacrificial templates method [74], and the silica nanoparticle sacrificial templates method [75, 76]. Among these, the silver nanoparticle sacrificial template method involves fabricating silver nanoparticles at a certain temperature and subsequently synthesizing HGNs by reducing chloroauric acid (HAuCl_4_) onto the silver nanoparticles [77, 78]. Similarly, HGNs composed of Au(III) grown on the surface of silica nanoparticles have been synthesized and characterized, with the silica cores ultimately removed through chemical reagents [75, 76]. Compared with the silver nanoparticle sacrificial template method, the cobalt nanoparticle sacrificial template method does not require high temperature, but its preparation conditions are relatively harsh. This method necessitates a fully anaerobic environment during synthesis to prevent the oxidation of the cobalt core, which is essential for obtaining a complete hollow sphere structure [79]. Recently, the cobalt boride sacrificial templates method has emerged as a novel design scheme for synthesizing HGNs [74]. The authors stressed that cobalt boride nanoparticles were utilized as sacrificial templates for the synthesis of HGNs, and the fabrication of cobalt boride sacrificial templates was different from before. Therefore, HGNs with varying particle sizes, porosity and optical properties for cancer nanomedicine were accurately obtained.

A few reviews on the HGNs have emerged so far [80–82]. Moreover, these reviews on the HGNs don’t discuss their synthesis methods and application in tumor treatment systematically. This review systematically introduced the preparation methods and the application of HGNs in cancer diagnosis and therapy. In addition, the issues that need to be addressed for a promising future of HGNs-based nanoplatform were also pointed out.

## Preparation of HGNs

### Silver nanoparticle sacrificial templates method

**Figure 1.**
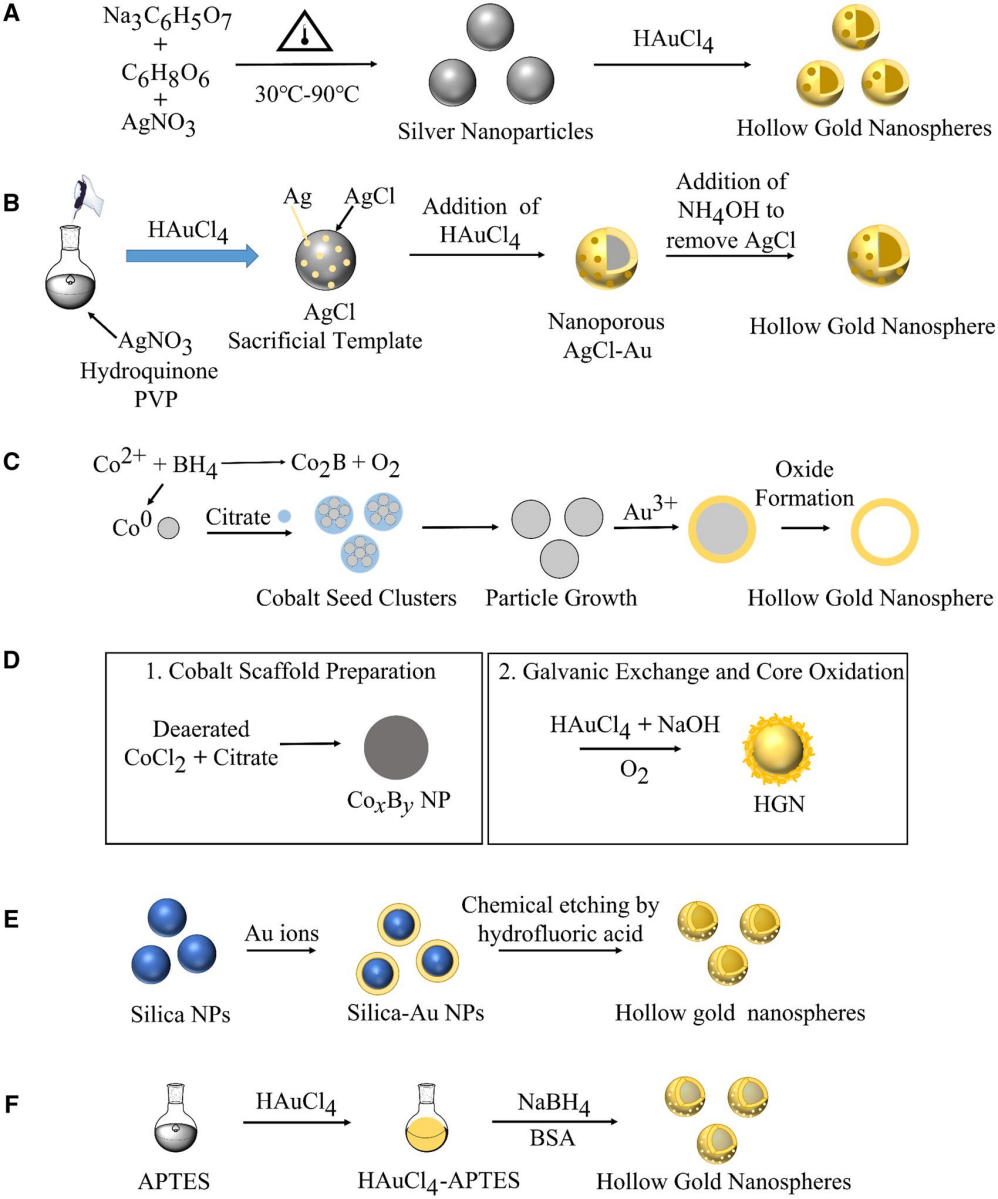
Synthesis of HGNs. (**A**) Silver nanoparticle sacrificial templates method [83]; (**B**) silver chloride nanoparticle sacrificial templates method [84]; (**C**) cobalt nanoparticle sacrificial templates method [85]; (**D**) cobalt boride nanoparticle sacrificial templates method [29]; (**E**) silica nanoparticle sacrificial templates method [86]; (**F**) one pot method [87].

The silver nanoparticle sacrificial templates method is one of the most widely used preparation methods for HGNs in recent years. The general procedure is based on the current exchange reaction between Au(III) and silver nanoparticles. Silver nanoparticles were first synthesized, and then HAuCl_4_ was reduced onto the silver nanoparticle templates. Subsequently, the silver nanoparticles were oxidized and dissolved in the solution under the coordination of NH_4_OH. Finally, Au/Ag nanospheres were obtained (Figure 1A).


$$3\text{Ag}+{\text{AuCl}}_{4}^{-}----\to \text{Au}\;+\;{3\text{Ag}}^{+}+{\;4\text{Cl}}^{-}$$

Ogunyankin *et al.* [83] synthesized the HGNs with diameters ranging from 10 to 40 nm by varying the reaction time and temperature using this method. In addition, the HGNs with a diameter of 10 nm exhibited an SPR range of 600–900 nm and a shell thickness of less than 2 nm. However, the morphology of HGNs was closer to a spherical shape only in small sizes and showed a cube shape in larger sizes. The authors also observed that nanobubbles triggered by NIR light around HGNs can transport proteins [88] and genetic materials [89, 90] into the cytoplasm [91] and even trigger the ablation of tumor cells [92, 93]. They ultimately concluded that the concentration of nanobubbles was closely related to the SPR peak of HGNs. Subsequently, Lukianova-Hleb *et al.* [94] then fabricated the more spherical HGNs using the silver nanoparticle sacrificial templates method. The diameter of HGNs fabricated in this study ranged from 50 to 70 nm and the thickness of the gold layer was 6–9 nm. Compared with Ogunyankin’s studies, these HGNs had a larger diameter and shell thickness, and their morphology tended to be more spherical. Moreover, through TEM images, the authors observed that the morphology of HGNs was irregular at low temperatures, which was due to the feverish temperature promoting the intraparticle ripening [87]. They also determined the gold element ratio of 86.28 ± 4.77% and silver element ratio of 11.24 ± 0.79% in HGNs using energy-dispersive X-ray spectroscopy line scans. It was proved that the HGNs were composed of not only Au element but also Ag element.

In addition, the HGNs alone exhibited low toxicity to cells, even at high concentrations. When the concentration of HGNs was not higher than 100 μg/ml, the viability of cells was slightly affected by short-time NIR irradiation with low power density, which indicates the HGNs had good biocompatibility and low biotoxicity. Depciuch *et al.* [86] then explored the influence of temperature on the size of HGNs during the preparation process. The results showed that the size of HGNs could be adjusted from 35 nm at 60°C to 76 nm at 90°C. Meanwhile, due to the porosity, SPR and photothermal conversion effects of HGNs were enhanced, the efficacy of PTT was also improved.

### Silver chloride nanoparticle sacrificial templates method

The silver chloride nanoparticle sacrificial templates method is an important method to prepare HGNs. In this method, the silver chloride sacrificial templates are formed upon adding HAuCl_4_ to the silver nitrate solution. Then, the Au(III) is reduced onto silver chloride nanoparticle templates under the cooperation of hydroquinone. Finally, the addition of NH_4_OH removes the silver chloride nanoparticle templates to obtain HGNs (Figure 1B).

Guo *et al*. [84] successfully synthesized HGNs using silver chloride nanoparticles as sacrificial templates. They first mixed a small amount of HAuCl_4_ with silver nitrate solution to obtain silver chloride nanoparticles, then added chloroauric acid drop by drop. In the presence of hydroquinone, the Au(III) was reduced to Au^0^ on the surface of silver chloride nanoparticles. Finally, the remaining silver chloride templates were removed by NH_4_OH. The size of HGNs could also be adjusted by altering the concentration of HAuCl_4_. In this study, the authors synthesized HGNs in a one-step process. The HGNs exhibited certain catalytic activity and stability in the reduction of 4-nitrophenol. Moreover, the catalytic activity remained even after storage for up to 1 month. The HGNs with catalysis activity prepared *via* this silver chloride sacrificial templates method might be promising candidates for future cancer nanomedicine.

### Cobalt nanoparticle sacrificial templates method

The cobalt nanoparticle sacrificial templates method has been widely used in recent years. The cobalt nanoparticles were formed *via* the reduction of Co(II) with NaBH_4_ in a deoxygenated environment. Then, HAuCl_4_ was added for the current exchange reaction. Finally, the cobalt templates were oxidized in an aerobic environment to obtain HGNs (Figure 1C).


$$3\text{Co}\;+\;{2\text{AuCl}}^{4-}----\to 2\text{Au}\;+\;{3\text{Co}}^{2+}\;+\;{8\text{Cl}}^{-}$$

Liang *et al.* [78] first successfully demonstrated cobalt nanoparticles could be used as sacrificed templates for HGNs synthesis in 2005. Pu *et al.* [85] prepared HGNs with various particle sizes using the cobalt nanoparticle sacrificial templates method. The authors adjusted the reaction temperature *via* water and ice bath and then bubbled nitrogen through the solution to ensure an anaerobic environment. The results showed that the diameter of HGNs (31.1–134.5 nm) was smaller than that of cobalt scaffolds (33.6–145.0 nm) within the reaction temperature range of 80–10°C. Meanwhile, the thickness of the gold shell was 5.4–18.4 nm in this reaction temperature range. The authors also found that the cobalt scaffolds solution changed from colorless to gray at low temperature (10–30°C) and turned brown at high temperatures (40–80°C) after the addition of NaBH_4_. The color of the solution would change accordingly in different reaction temperatures. In this study, the influence of cobalt scaffolds at various reaction temperatures on the size and shell thickness of HGNs were also investigated. Methods for preparing HGNs with different sizes were provided, and new design strategies were proposed for drug delivery using HGNs as well.

According to the TEM images, the morphology of HGNs was spherical, resulting from the cobalt nanoparticle sacrificial templates method [72, 73, 95]. Preciado-Flores *et al.* [96] also found that when polyvinyl pyrrolidone (PVP) was used as a stabilizer, the HGNs would aggregate into chains, which weakened their dispersion and affected the application of the HGNs. While some HGNs exhibited irregular morphology, the improvement in morphology was evident in comparison to the silver nanoparticle sacrificial templates method.

### Cobalt boride nanoparticle sacrificial templates method

The cobalt boride nanoparticle sacrificial templates method is a novel approach to synthesize HGNs based on cobalt nanoparticle sacrificial templates. This method is quite similar to the cobalt nanoparticle sacrificial templates method, except that the sacrificial templates are cobalt boride nanoparticles. In addition, boron hydrogen acid (B(OH)_4_^−^), the final hydrolysate of NaBH_4_, also aids the HGNs synthesis. The principle is as follows: under anaerobic conditions, Co(II) is reduced to cobalt boride nanoparticles by NaBH_4_ and B(OH)_4_^−^, and subsequently, the surfaces of cobalt boride nanoparticle templates are oxidized upon adding HAuCl_4_. The remaining templates are completely oxidized under aerobic conditions to form HGNs (Figure 1D).


$$\begin{array}{l} {2\text{CoCl}}_{2}\;+{\;4\text{NaBH}}_{4}\;+----\to {\;9H}_{2}{O\;\text{Co}}_{2}B\;+\;4\text{NaCl}\;+\;12.{5H}_{2}\;+\;3B{\left(\text{OH}\right)}_{2}\\ {4\text{Co}}_{2}B\;+{\;3O}_{2}----\to 8\text{Co}\;+{\;2B}_{2}O_{3}\\ {3\text{Co}}^{0}\;+\;{2\text{AuCl}}_{4}^{-}----\to {3\text{Co}}^{2+}\;+\;{2\text{Au}}^{0}\;+\;{8\text{Cl}}^{-} \end{array}$$

Lindley *et al.* [29] synthesized HGNs using cobalt boride nanoparticles. They reduced Co(II) with B(OH)_4_^−^ and ${\text{BH}}_{4}^{-}$ to produce boride cobalt nanoparticles, utilizing sodium citrate as a stabilizer. The reaction of HAuCl_4_ with cobalt boride nanoparticle templates led to the formation of a gold shell. The HGNs were obtained by oxidizing the remaining templates under the aerobic condition. In the preparation of HGNs, the authors adjusted the aerobic and anaerobic conditions, the ratio of B(OH)_4_^−^ and ${\text{BH}}_{4}^{-}$, and the proportion of NaBH_4_ and Co(II). The HGNs with the same diameter but different SPRs or different diameters but the same SPR were discovered. In addition, the HGNs produced under aerobic or anaerobic condition showed a significant difference in morphology. When the molar volume of HAuCl_4_ was 0.3 μmol, the shells were discontinuous and appeared to be pieced together by smaller HGNs in aerobic conditions. However, the Au shells were smoother and more complete under anaerobic conditions. Moreover, the SPR of HGNs was stronger and relatively red-shifted (760 nm) under anaerobic conditions compared with that of HGNs under aerobic conditions (665 nm).

Therefore, Lindley *et al.* [97] further explored the influence of oxygen on the characteristics of HGNs. In the presence of oxygen, the authors adjusted pH using NaBH_4_ to change the surface morphology and obtained HGNs with a rough surface. The results showed that the photothermal effect of HGNs with rough or smooth surfaces were similar, while the rough HGNs maintained good photothermal cycle characteristics and might be widely applied in drug delivery and catalysis for cancer nanomedicine. This method provided a new strategy for the preparation of HGNs. The drug loading capacity of HGNs improved with the increase in surface roughness, so the HGNs were potential as nanocarriers mediated multimodal therapy based on PTT.

### Other preparation methods

Other methods utilize non-metallic materials as templates for synthesizing HGNs. For example, Guan *et al.* [98] synthesized HGNs directly using a one-pot method. The authors mixed HAuCl_4_ with 3-aminopropyltriethoxysilane (APTES) suspension, using bovine serum albumin (BSA) as a stabilizer, and then reduced Au(III) with NaBH_4_ to obtain HGNs. In addition to the simple preparation process, the photothermal effect and stability of the HGNs have also been improved. Moreover, Nia *et al.* [99] fabricated HGNs using secondary gold resources as raw materials and silica nanoparticles as templates. The authors first produced gold in the presence of copper anode slime, then purified gold after washing it with concentrated nitric acid. The silica nanoparticles were utilized to form the core-shell structure. Finally, HAuCl_4_ was added to remove the silica templates, resulting in the obtainment of HGNs. This preparation method facilitates the recovery of gold, which aligns with the concept of green chemistry.

Each of the preparation methods is summarized below (Table 1). The shell of the silver nanoparticle sacrificial templates method is made of a gold and silver alloy, rather than just gold when compared to the silver chloride nanoparticle sacrificial templates method. As for the cobalt boride nanoparticle sacrificial templates method, it is found that the surface roughness of HGNs can be adjusted in the presence of oxygen. Thus, the HGNs serving as nanocarriers will be promising for cancer multimodal therapy.

**Table 1.** Summary of preparation methods of HGNs

Preparation method	Mean particle size (nm)	Shell thickness (nm)	SPR peak (nm)	Advantages	Disadvantages	Ref.
Silver nanoparticle sacrificial template method	10–40	1–3	600–900	Small particle size HGNs can be prepared at low temperatures	The morphology of HGNs is irregular	[83]
	50–70	6–9	780 (maximum)			[94]
	35–76	–	500–900			[86]
	54.8 ± 1.6	–	808 (maximum)			[100]
Silver chloride nanoparticle sacrificial template method	80–350	–	584–664	One-step synthesis of HGNs	The prepared HGNs contain Ag impurities	[84]
Cobalt nanoparticle sacrificial template method	31.1–134.5	5.4–18.4	565–850	It is widely used, and the spherical structure of the prepared HGNs is obvious	HGNs tend to aggregate into chains with PVP as a stabilizer	[85]
	40.5 ± 7	–	500–900 (HGNs)			[101]
	83\74\73\83	29\21\12\11	620\680\786\790 (maximum absorbance of HGNs)			[102]
	–	–	780–800 (maximum)			[103]
	46 ± 4.8	4.0 ± 0.3	798 (maximum)			[104]
	61.44	∼7	500–700			[105]
	∼78	–	616 (maximum)			[79]
Cobalt boride nanoparticle sacrificial template method	37\48\73	–	600–900	Proposed borohydride as a new growth agent, and prepared HGNs with different surface roughness	The experimental conditions are harsh and difficult to control	[74]
	56–90	10–44	650–950			[97]
One pot method	37–96	∼10	657–957	Simple preparation process	Poor dispersion	[98]
Silica sacrificial template method	100	10	400 (maximum)	Both porosity and active specific surface area have increased	The morphology and porosity are irregular	[99]

## HGNs-based nanocomposites for cancer treatment

### Photothermal therapy

It is well known that HGNs possess unique hollow structures. Meanwhile, due to SPR properties, HGNs also show intense light absorption performance. The absorbed light energy of HGNs is further converted into thermal energy to achieve PTT [52, 106]. However, the penetration and retention of HGNs alone *in vivo* are not sufficient to exert their properties. Therefore, Encabo-Berzosa *et al.* [101] introduced mesenchymal stem cells (MSCs) to enhance the stability of HGNs *in vivo*.

Due to the properties of cells themselves, MSCs can also migrate and bind to sites of inflammation, including tumor cells [107–109]. In this study, the size of HGNs with cobalt nanoparticles as templates was 40.5 ± 7 nm. The localized SPR of pegylated HGNs (PEG-HGNs) showed a slightly blue shift compared with that of HGNs and apparent absorption in the NIR region. Meanwhile, the toxicity of PEG-HGNs significantly decreased, which was also consistent with previous studies [110]. After peritumoral injection *in vivo*, the authors found that the tumor size of mice treated with MSCs **+** HGNs decreased by approximately 80% within 12 days after 808 nm laser irradiation (1 W cm^−2^, 5 min), but was still larger than that treated with HGNs alone. This was due to the fact that only a portion of HGNs was internalized into MSCs during incubation, with the possibility of even losing partial HGNs during cell washing. After intravenous injection of PEG-HGNs, dark spots were recognized in tumor areas, but not in the mice of the control group. It was also a strong proof that metal nanoparticles could rapidly accumulate in tumor areas *in vivo* [111, 112]. In addition, 12 days after laser irradiation, an obvious reduction in tumor size was observed in the group injected with MSCs+PEG-HGNs compared with the other groups. Therefore, a new strategy for the efficient delivery of HGNs was provided. The MSCs not only improved the stability of HGNs and made it difficult for HGNs to be detected or phagocytized by reticuloendothelial system (RES) macrophages during the circulation *in vivo*, but also had the characteristic of accumulate in the tumor region, thus providing a new strategy for PTT.

In addition to passive tumor targeting, receptor-based active targeting using HGNs-based nanoplatform is another promising system for cancer therapy. Although the MSCs exhibit the characteristics of migration to the tumor region, they still cannot ensure that the HGNs are precisely transported into tumor cells. Subsequently, to realize the active targeting-mediated cancer therapy of the HGNs-based nanomaterials, Vickers *et al.* [102] also conjugated folic acid (FA) onto the HGNs. The purpose of the study was to explore the two-photon photoluminescence and photothermal properties of HGNs. The FA-HGNs achieved efficient PTT with a shorter irradiation time and lower laser power density (39 mW). Moreover, the HGNs displayed better photothermal effects than gold nanorods. Additionally, the anti-growth factor antibody C225 provided an active targeting mechanism to HGNs thus enhancing the photothermal effect of HGNs [113]. In the study, the authors investigated whether the short pulse of irradiation could selectively destroy tumor areas or kill tumor cells. They studied the cellular distribution in A431 tumor cells and found that more than half of the C225-HGNs (54 ± 14%) were attached to the cell membrane, while the rest were distributed in the cytoplasm. After the first laser pulse, the color of C225-HGNs changed from green to brown, indicating the collapse of the hollow structure of HGNs. Moreover, A431 cells pretreated with C225-HGNs were significantly damaged after just two laser pulses, while untreated A431 cells still survived.

In addition to tumor membrane targeting, nucleus targeting is also a desirable choice for PTT of HGNs [100]. Compared to normal cells without the receptors, HGN-AS1411 exhibited greater targetability towards A375 tumor cells with the receptor. The results demonstrate that active targeting-mediated PTT of HGNs is a promising therapeutic strategy.

### Photothermal immunotherapy

As is well known, PTT can effectively kill tumor cells through laser irradiation. With the development of NIR irradiation, PTT shows obvious advantages in treating original or metastatic tumors. Despite this unique feature, it is still difficult for PTT to kill tumor cells completely, which limits its application in cancer nanomedicine. Over the past few years, immunotherapy has become an attractive strategy among different treatment options and has shown its prospect against malignancies. It induces an anti-tumor response that can defeat cancer with the aid of the body immune [114]. Nevertheless, the impaired immune monitoring system leads to cancer immune tolerance, which is the main obstacle to inducing anti-tumor immune response. Therefore, photothermal immunotherapy was introduced to kill tumor cells with laser irradiation, but also stimulate the autoimmune system to eliminate the residual tumor cells [115]. PTT was reported to stimulate the expression of heat shock protein 70 (Hsp70) in cancer cells and promote cytotoxic lymphocytes to kill tumor cells [116, 117]. With the help of immune adjuvant, PTT can also promote anti-tumor immune responses which was shown as a strategy for the treatment of metastatic tumors [118]. Therefore, NIR laser-mediated photothermal immunotherapy may be a potential method for cancer treatments to kill tumor cells completely.

Immune checkpoint therapy has been a hot immunotherapy strategy topic in recent years [119]. This method restores the function of T cells by blocking inhibitory checkpoints or activating them with therapeutic antibodies to stimulate autoimmunity, thereby reducing the uptake of chemotherapy drugs [120]. Although PD-1 inhibitors have lower costs and less immunotoxicity, they are still easily cleared *in vivo*, requiring frequent administration in cancer therapy [121–123]. In order to maintain the immune system in a state of long-term activation, Luo *et al.* [124] created a poly(lactic-co-glycolic acid) (PLGA) nanoplatform coated with HGNs and anti-PD-1 peptide (APP). Upon irradiation of NIR light, the HGNs generated heat that killed tumors *in situ*. The heat further expedited the release of anti-PD-1 agents that killed tumor cells *in situ* even distant ones. Additionally, they introduced Cytosine-phospho-guanine (CpG), an immune adjuvant, allowing the nanoparticles to act as vaccines [125]. At the same time, Luo *et al.* [126] directly connected CpG to HGNs to obtain a nanomaterial with a smaller particle size, which was more advantageous for the effective accumulation of CpG in the tumor area *via* body circulation and played an effective role in immunotherapy. Immunotherapy can not only restore the function of T cells by blocking inhibitory checkpoints but also stimulate the immune response, such as T cell proliferation and dendritic cells (DCs) antigen delivery. Nowadays, various treatments have also been applied to immunogenic cell death (ICD), such as chemotherapy [127], radiotherapy [128], photodynamic therapy (PDT) [129], etc. Among them, PDT is particularly promising in cancer therapy because reactive oxygen species (ROS) stimulate signaling pathways in cells to affect ICD. However, these danger signal factors only exist in the endoplasmic reticulum (ER) of the cell, and most photosensitizers (e.g. indocyanine green (ICG)) are distributed in the cytoplasm after cell internalization, which limits the application of PDT.

**Figure 2.**
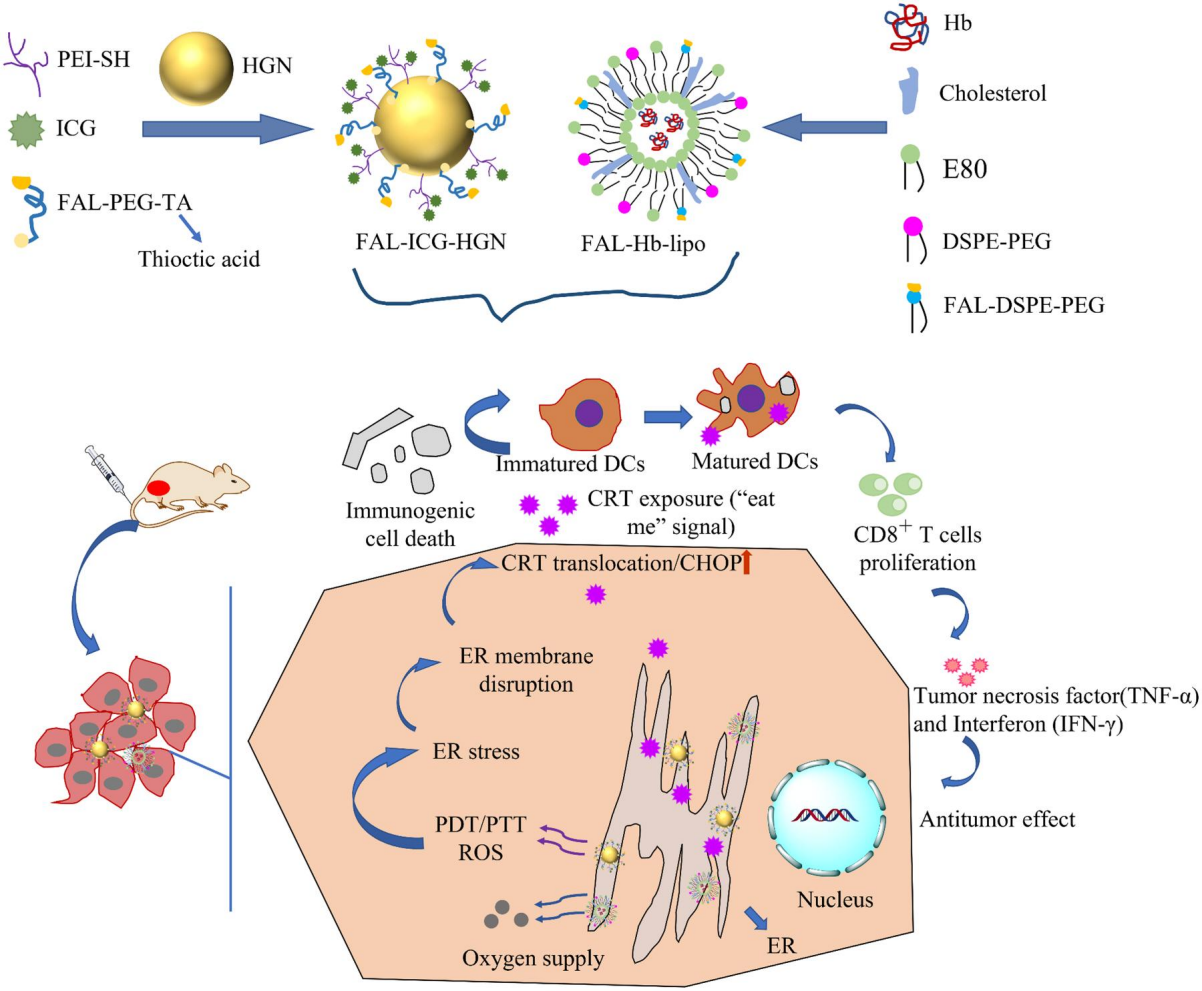
A nanoplatform consists of ER-targeting pardaxin peptides (FAL) modified-, ICG conjugated-HGN (FAL-ICG-HGN), together with an oxygen-delivering hemoglobin (Hb) liposome (FAL-Hb lipo), designed to realize PTT/PDT/immunotherapy under the irradiation of NIR light [103].

Therefore, ER-targeting pardaxin (FAL peptide) modified HGNs encapsulating ICG were designed for drug delivery in PDT-PTT-promoted ICD-associated immunotherapy [116]. Meanwhile, in order to improve the hypoxic environment within tumor cells, hemoglobin (Hb), an efficient and safe oxygen carrier, was also introduced, providing a new strategy for PDT (Figure 2). The authors prepared FAL-ICG-HGNs and FAL-Hb-lipo with a nanoparticle size smaller than 200 nm. The CHOP protein, C/EBP-homologous protein-10, produced by ER stress, showed significantly up-regulated after irradiation in the FAL-ICG-HGNs plus FAL-Hb-lipo group. Simultaneously, they found that FAL-targeted PDT could promote the translocation of calreticulin (CRT, a maker for ICD, located in ER) to the cell membrane. This result was also proven *in vivo*. Moreover, the FAL-ICG-HGNs plus FAL-Hb-lipo group displayed a higher maturation rate (33.1%) of tumor DCs than the other groups. This also confirmed the mechanism: the ROS generated *via* PDT promoted the occurrence of CRT, while the membrane transport of CRT was stimulated under irradiation. Subsequently, the combination of CRT and DCs stimulated DCs to present antigens to activate the adaptive immune response, that is, to form CD8^+^ T cells which secreted toxic substances against tumor cells. In addition, the anti-tumor effect of mice without CD4^+^ and CD8^+^ T cells was not obvious compared with the mice with T cells after being treated with FAL-ICG-HGNs plus FAL-Hb-lipo. This study illustrated the photothermal immunotherapy for cancer treatment and provided a new approach for other immunotherapy-based multimodal therapy.

### Chemo-photothermal therapy

Chemotherapy is a traditional treatment method that inhibits the proliferation of tumor cells using chemotherapeutic drugs. However, frequent chemotherapy causes drug resistance in tumor cells and some damage to normal tissues. Chemo-PTT has been widely applied in cancer therapy in recent years. This treatment can eliminate tumor cells completely with less damage to normal tissues.

Two HGNs-based nanoparticles have been synthesized for chemo-PTT [130]. Zhou *et al.* first prepared HGNs using cobalt nanoparticles as templates. One strategy involved first oxidizing the cobalt nanoparticle and then binding DOX onto HGNs *via* electrostatic interaction. The other method was to add DOX·HCl into the reaction medium during the synthesis process, and DOX was precipitated within the hollow structures of HGNs. It was demonstrated that the photothermal conversion efficiency and biotoxicity of HGNs with DOX precipitated in the hollow structures were greater than those of HGNs prepared through electrostatic interaction. In this study, the authors comprehensively introduced a new strategy to prepare DOX-loaded HGNs. However, DOX-HGNs were injected into mice at intratumor injection, which could only treat epidermal tumors. The active targeting agents have been utilized to target deep-seated tumors via intravenous injection of DOX-loaded HGNs for cancer therapy.

The human epidermal growth factor receptor-2 (HER-2) antibody served as targeting ligands to endow nanoparticles with the property of active targeting [131]. In this study, liposomes were utilized as nanocarriers. HGNs and DOX were loaded into the liposomes. Then the liposomes were modified with HER-2 antibody. The average diameter of the HGNs and DOX-loaded targeting liposomes was 154.7 ± 2.9 nm. On the 14th day after intravenous injection, the tumor weight of mice showed a significant decrease after the treatment with HGNs and DOX-loaded targeting liposomes plus laser, using a lower dose of DOX and weaker power density than those in their previous studies. Compared with the liposomes mentioned above, the hydrogels were also used as drug carriers to load DOX and HGNs [132]. The difference was that DOX was not only encapsulated in hydrogels but also attached to the surfaces and cavities of HGNs. Therefore, the DOX could be released continuously and slowly, which enhanced the treatment effect. In recent years, imaging tools have been widely used in tumor treatment, too. Wang *et al.* [133] studied ultrasound imaging-mediated combination therapy utilizing HGNs. They prepared micelles loaded with PTX and HGNs, using TNYL peptides as targeting ligands. Based on fluorescence imaging, they demonstrated the feasibility of targeting the micelles in the tumor area and performed precise laser irradiation on the tumor region to enhance the PDT effect. In addition, the authors prepared DOX-loaded HGNs functionalized with targeting agents and carried out *in vivo* studies. They concluded that the nanoparticles could inhibit tumor growth as well [104]. Therefore, HGNs could be used not only as photothermal materials but also as nanocarriers for drug delivery. In addition to DOX, HGNs could also load other chemotherapeutics for chemo-PTT. Shen *et al.* [134] synthesized docetaxel (DTX)-loaded HGNs with FA as targeting agents. The authors concluded that the HGNs did have remarkable targeting to tumor cells and excellent inhibitory effect on the growth of tumor cells, demonstrating an obvious therapeutic effect. Protopanaxadiol, which had inhibitory effects on a variety of tumor cells, was loaded onto HGNs to improve their poor water solubility [135]. After studying the cell viability *in vitro* and testing the tumor inhibitory effect, the authors concluded that the HGNs effectively promoted the apoptosis of tumor cells and inhibited tumor growth. Guan *et al.* [136] also loaded cisplatin as a chemotherapeutics onto HGNs, which showed high cytotoxicity to tumor cells and could effectively inhibit tumor growth *in vivo*.

### Photothermal and photodynamic therapy

PDT has captured much attention in recent years. The general process is based on photosensitizers, which can effectively generate ROS and kill tumor cells after irradiation. PDT not only has the properties of generating ROS but also shows few side effects on normal tissues [137–141]. However, many factors also limit the application of photosensitizers, such as poor water solubility, easy to be quickly removed *in vivo*, lack of targeting, etc. [142–144]. Moreover, the ROS generated by PDT also stimulates the resistance of the antioxidant balance system in tumor cells, thus weakening their therapeutic effect [145, 146]. Therefore, the HGNs selected as nanocarriers can exert the effect of PTT and be combined with PDT, thereby mitigating the issue of uneven heating during PTT and ultimately leading to the elimination of tumor cells.

**Figure 3.**
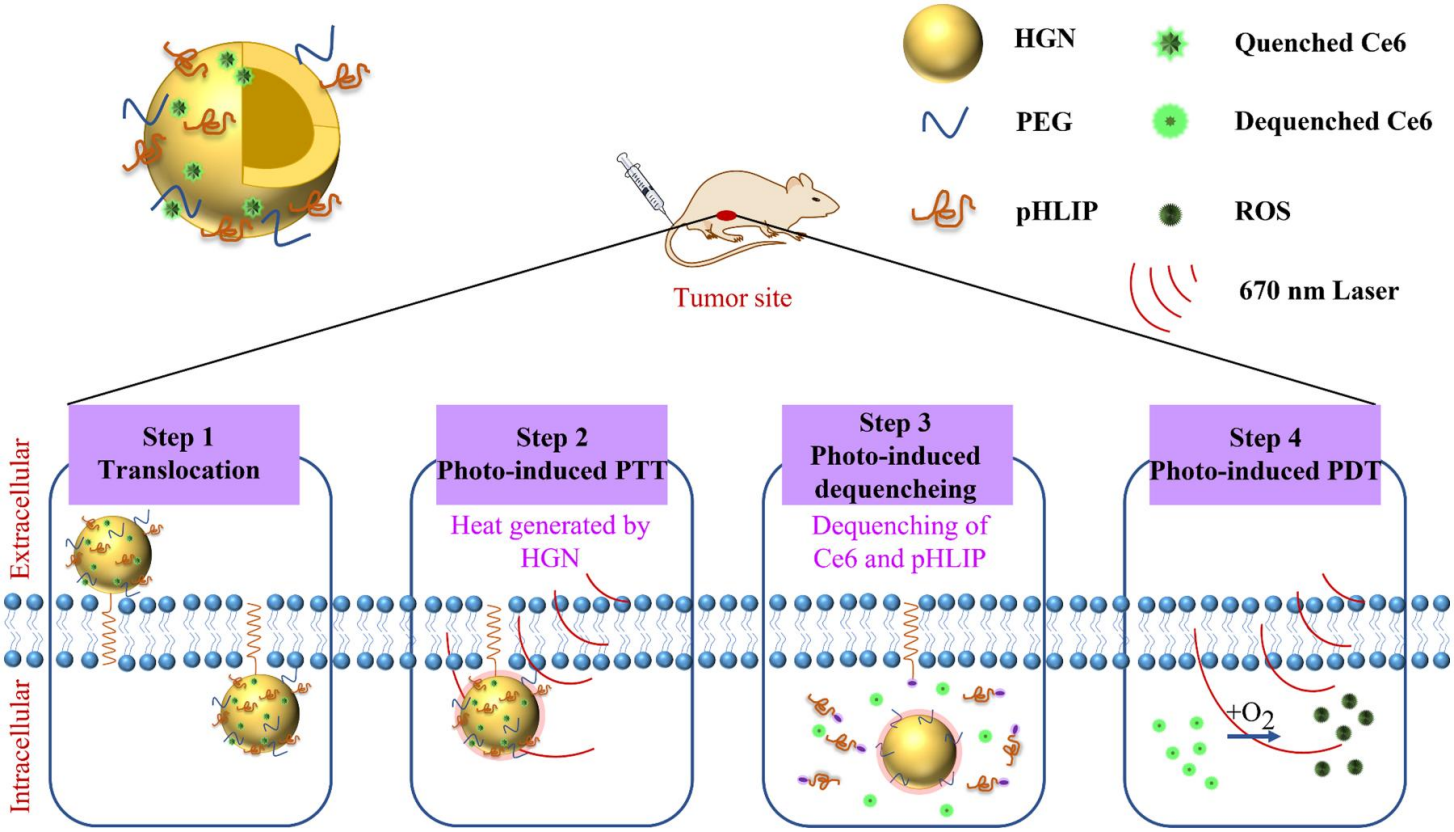
HGNs link pH (low) insertion peptides (pHLIP) and photosensitizers (Ce6) for active target-mediated PPT/PDT. The HGNs serve as photothermal sequesters for receiving the fluorescence of Ce6, thereby resulting in the fluorescence disappearing of Ce6, are also introduced [140].

Chlorin E6 (Ce6), a photosensitizer, showing strong absorption peaks at wavelengths of 405 and 660 nm, and strong fluorescence emission at 668 nm, was reported to be used for synthesizing the nanoparticles (HGNs-pHLIP-Ce6) to achieve pH-driven targeting PTT/PDT. The laser (660 nm) was applied to excite Ce6 to produce ROS, which executes the PDT effect. With HGNs served as photothermal coupling agents, Ce6 was temporarily quenched *via* fluorescence resonance energy transfer (FRET), leading to non-fluorescence and non-phototoxicity during the circulation after intravenous injection. While delivered into tumor tissue, Ce6 released from HGNs under the irradiation of 660 nm, along with partial degradation of Ce6 for ROS generation. Therefore, Ce6 dequenched and showed fluorescence for the real-time tracer. The targeting pH-insertion linking peptide (pHLIP) was bound to HGNs *via* electrostatic interaction, which had been proved to be rapidly inserted into the lipid bilayer of tumor cells and then transferred the binding nanoparticles into the cells, facilitating pH-responsive cell internalization (Figure 3) [140]. Meanwhile, HGNs have been demonstrated to serve as photothermal sequesters, which received fluorescence from suppository organic dyes (e.g. photosensitizers) *via* fluorescence resonance energy transfer, so that the fluorescence of these dyes temporarily disappeared. The HGNs-pHLIP-Ce6 nanoparticles-mediated PTT/PDT can be performed simultaneously under a single laser beam, which simplifying the treatment process. The HGNs-pHLIP-Ce6 also showed excellent biotoxicity and tumor growth inhibition than free Ce6. Moreover, 24 h after injecting HGNs-pHLIP-Ce6 intravenously, most of the Ce6 accumulated in the tumor region, rather than in the liver or other metabolic organs.

The ICG can be also served as photosensitizers covalently bonded to HGNs. This covalent bond was more stable than the electrostatic binding mentioned above, preventing the loss of the drugs during circulation [147]. Under the 808 nm irradiation, ICG-HGNs showed a stronger photothermal effect than that of the HGNs alone, and the cell viability was also lower, due to the heat generation of HGNs and both heat/ROS generation from degradable ICG. In addition to this, ICG showed an excitation wavelength of 785 nm and an emission wavelength of 810 nm, and could be used for fluorescence imaging *in vitro* or *in vivo*. After injecting ICG-HGNs intravenously, plentiful fluorescent accumulation was observed in the tumor area, and tumor growth in mice was significantly inhibited, as well as all mice survived up to 40 days. In addition, the TNYL peptide was used to enhance the stability of the ICG-HGNs *in vivo*, actively targeting the EphB4 receptor of tumor cells to achieve more precise drug delivery [148].

However, the ROS generated by PDT stimulated the antioxidant balance system of tumor cells, leading to photodynamic resistance, which further limited the effect of PDT [145, 146].

**Figure 4.**
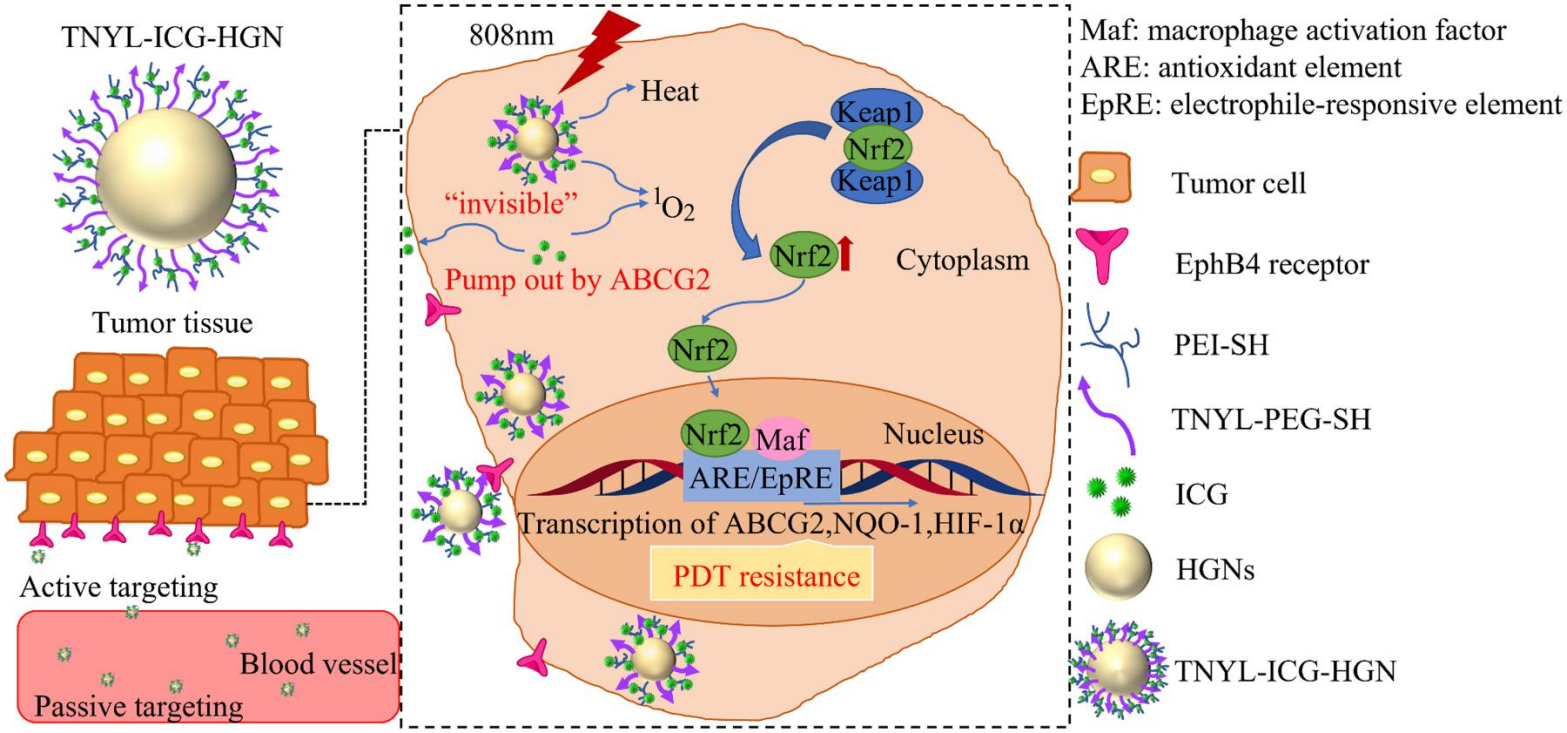
Tumor cells targeting TNYL-ICG-HGNs nanoparticles with ABCG2 escape function were prepared. The expression of the intracellular antioxidant response factor Nrf2 is regulated by ROS generated ICG under the NIR irradiation, thereby eliminating the anti-PDT effect of tumor cells [148].

Therefore, the mechanism of anti-PDT in drug delivery for cancer therapy was also investigated (Figure 4). Factors in anti-PDT, namely ATP-binding cassette transporter (ABCG2), NAD(P)H: quinone oxidoreductase 1 (NQO-1) and hypoxia-inducible factor-1α (HIF-1α), are regulated by nuclear factor erythroid 2-related factor 2 (Nrf2, which induced an antioxidant response and activated by keap1). The expression of Nrf2 treated with free ICG was downregulated after the second round of irradiation, while rose to a normal level after the third round of irradiation. After 3 rounds of irradiation, compared to the free ICG, the expression of Nrf2 in tumor cells was significantly downregulated when treated with the TNYL-ICG-HGNs. ABCG2 maintained the homeostasis of tumor cells and regulated the cellular accumulation of various drugs to achieve anti-PDT. Due to the presence of the free ICG, the tumor cells immediately stimulated the generation of ABCG2, thereby eliminating ingested ICG. In contrast, the expression of ABCG2 treated with TNYL-ICG-HGNs was relatively low and returned to normal levels after several rounds of irradiation. This also demonstrated that the ICG in the TNYL-ICG-HGNs showed the ability to escape ABCG2, thereby reducing the anti-PDT effect of TNYL-ICG-HGNs. NQO-1 expressed the level of ROS in cells. The NQO-1 remained high when treated with TNYL-ICG-HGNs after irradiation. After irradiation, the level of HIF-1α, which is expressed under hypoxic conditions and controlled by Nrf2, underwent a transition from increasing to decreasing when treated with free ICG or TNYL-ICG-HGNs. In the end, the level of HIF-1α treated with free ICG was similar to that in the control group, while it was significantly lower when treated with TNYL-ICG-HGNs. The authors speculated that TNYL-ICG-HGNs might cause the collapse of the Nrf2-regulated anti-PDT mechanism after irradiation, thus presenting an extraordinary antitumor effect.

From the above, the TNYL-ICG-HGNs showed good stability and obvious anti-tumor effect on general anti-PDT tumor cells. Moreover, the introduction of TNYL peptides also enhances the accumulation of the drugs in the tumor tissues. We speculate that other photosensitizers could overcome the photodynamic resistance using a similar strategy.

### Other tumor treatments

In addition to the aforementioned therapy, the scholars also studied other treatment methods for HGNs. For instance, Deng *et al.* synthesized HGNs-based nanoparticles to realize multimodal therapy (chemo/photothermal/photodynamic therapy). They prepared DOX-loaded HGNs, whose surfaces were modified with photosensitizer Ce6. After laser irradiation, HGNs showed a photothermal effect, Ce6 generated ROS, and DOX demonstrated chemotherapy effects on tumor cells, thereby achieving multimodal therapy [149].

Moreover, the FAL-ICG-HGNs plus FAL-Hb-lipo we introduced above also achieved photothermal/photodynamic/immune therapy [121]. To realize photothermal gene therapy, Hu *et al.* [150] modified pDNA onto the surface of HGNs, directly inhibiting tumor growth by controlling gene transfection in the tumor cells.

## HGNs-based nanosystem for imaging-guided tumor therapy

In recent years, therapeutic drugs with functions of imaging and treatment have attracted enormous attention in cancer diagnosis and treatment. Among these, HGNs are highly potential for the development of theranostic agents in cancer diagnosis. Photothermal imaging has been demonstrated to show the real-time temperature change image of contrast agents. Under the irradiation of NIR, HGNs can convert the light into heat energy and be observed using the infrared thermal imaging camera. On the other hand, due to the minimal absorption of human tissues and water in the range of NIR, the HGNs with strong NIR absorption can be chosen as the contrast agents for photoacoustic imaging, which is a noninvasive imaging modality combined with the high selectivity of optical imaging and high spatial resolution of ultrasonic imaging within a depth of centimeters. In addition to this, HGNs show highly promising drug nanocarriers with the unique cavity structure. Various imaging agents are available for covalent or non-covalent into HGNs to realize precise diagnosis and treatment. Combined with the imaging agents and drug nanocarriers, HGNs will have great potential for application in the biomedical field.

### HGNs-based nanoplatforms for ultrasound imaging-guided tumor therapy

Ultrasound imaging utilizes an ultrasonic beam to scan the human/animal body and obtains the images of organs by receiving and processing the reflected signals.

Most studies of photothermal-chemotherapy in cancer employ conventional subcutaneous xenograft tumor models [151]. However, tumors better reflect the original tumor microenvironment when tumor cells are implanted directly into the organ *in situ*, rather than in subcutaneous xenograft tumor models [152, 153]. To date, photothermal-chemotherapy has rarely been demonstrated in orthotopic tumor models and it is challenging to provide deep orthotopic tumors with precise irradiation. To accurately observe the HGNs-based nanoparticles in tumors, ultrasound imaging was introduced [132]. The HGNs performed effective photothermal conversion efficiency under NIR light, enabling PTA in tumor area. In this study, the HGNs-based micelles could not only actively target tumor cells, but they also showed great inhibition of orthotopic clone tumors. Moreover, the PTA in tumor could be directly observed before and after NIR irradiation under the guidance of ultrasound imaging.

For ultrasound imaging-guided cancer therapy, it is a top priority to find superior ultrasound contrast agents. ICG served as a contrast agent for ultrasound imaging in the study mentioned above, where it was labeled onto nanoparticles for use in ultrasound imaging. Perfluorocarbon (PFC) was also a nano-sized ultrasound contrast agent but exhibited lower echogenicity due to insufficient accumulation in tumor sites. Therefore, the liposomes were introduced as nanocarriers to load DOX, HGNs and PFC (DHPL) for chemo-PTT [154]. The heat generated by HGNs under the NIR irradiation triggered the gasification of PFC, significantly enhancing the ultrasonic signal. In addition, it was noted that the cytotoxicity of DHPL showed significant enhancement under NIR irradiation due to the heat-induced size change of DHPL and the release of DOX. Nevertheless, the HGNs could also be utilized as nanocarriers in addition to being photothermal agents. Perfluorohexane (PFH), an ultrasound contrast agent and oxygen transport agent, and O_2_ were loaded into HGNs to obtain oxygen self-enriched nanosystems [155]. Based on delivered O_2_, the DNA damage repair was inhibited by cisplatin which led to cell apoptosis. Furthermore, the anti-tumor effect of the HGNs-based nanoplatform was significantly improved, and the disintegration of tumor cells was also observed with ultrasound imaging. Moreover, Shang *et al.* [156] prepared the polydopamine loaded HGNs, which showed an enhanced ultrasound imaging signal, and the photothermal effect on tumor cells was also obvious.

### HGNs-based nanoplatforms for Raman imaging-guided tumor therapy

Raman imaging technology is a new generation of fast, high-precision, 2D-scanning laser Raman technology, boasting characteristics such as high-speed and extremely high-resolution imaging.

Nagy-Simon *et al.* [157] successfully synthesized Ag/Au hollow nanospheres (HNS) with NIR-responsive properties. The surfaces of HNS were modified with anti-CD19 monoclonal antibodies and Nile Blue. It was demonstrated that the modified HNS were successfully internalized into the cancer cells *via* Raman imaging and two-photon excited fluorescence imaging. The imaging results proved that the modified HNS were promising agents for specific targeting cancer therapy guided by Raman imaging. In addition to Nile Blue, other Raman scattering nanoprobes have also been used for cancer therapy. Liu *et al.* [158] covalently modified HGNs with Raman-active azide derivatives (DNBA-N_3_) to obtain single-layer Raman tags on the surfaces of HGNs and strong surface-enhanced Raman scattering (SERS) signals. The SERS probe DNBA-N_3_ showed good stability and biocompatibility. Meanwhile, the DNBA-N_3_-modified HGNs were conjugated with FA and exhibited obvious cell internalization [159–162].

In recent years, the approaches of NIR-sensitive SERS nanoprobes have been presented. The NIR-sensitive SERS nanoprobes were first labeled onto Ag/Au HNS hollow nanospheres, which were subsequently connected to the surfaces of silica nanoparticles [163]. The signal of the HNS-based nanocomposites was 100 times stronger than that of non-resonant molecular-marked HGNs, which illustrated promising ways to detect the nanoparticles accurately.

### Nanomaterials based on HGNs for fluorescence imaging-guided tumor therapy

The fluorescence signal intensity emitted by fluorescent substances after excitation is linearly related to their amount within a certain range.

**Figure 5.**
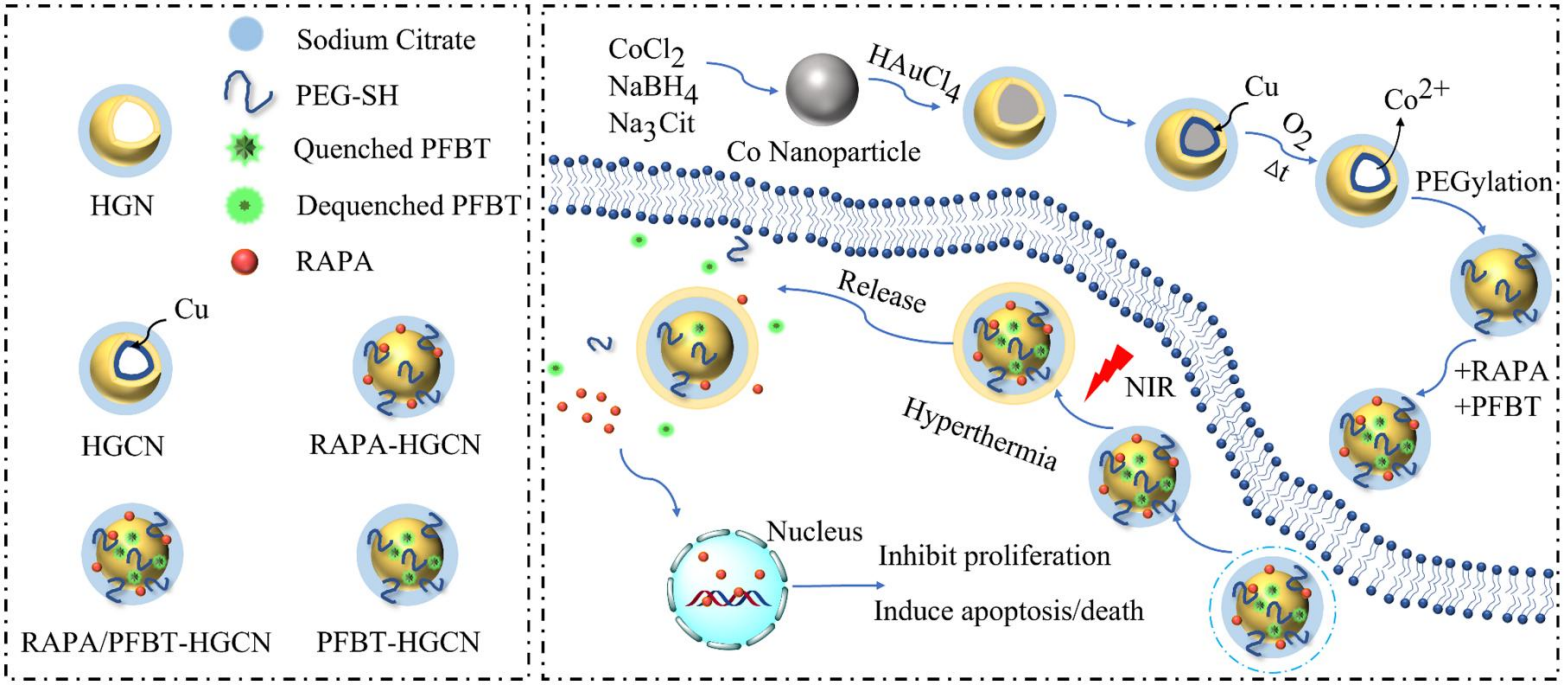
PFBT was used to monitor the distribution of RAPA/PFBT-HGCNs in real-time in cells or *in vivo* with the guidance of fluorescence imaging. The PFBT was detached from the surface of HGCN by NIR irradiation, thereby re-generating the fluorescence received by HGCN, and achieving the tumor diagnosis and treatment [105].

To adequately recognize the tumor site and precisely irradiate nanoparticles within the tumor, the presentation of fluorescent probes with the property of real-time tracing is essential for cancer therapy. Therefore, a new hollow Au-Cu nanoplatform (RAPA/PFBT-HGCNs) was developed [105]. The contrast agent fluorescent dot polymer (9,9-dioctylfluorene-2,7-diacyl-benzothiazole) (PFBT) and the rapamycin (RAPA, an mTOR inhibitor to block tumor growth) were loaded into HGNs for real-time NIR fluorescence tracing and cancer therapy (Figure 5). Among them, the HGNs were introduced to serve as photothermal sequesters after absorbing PFBT, with the ability to receive the fluorescence of PFBT *via* the fluorescence resonance energy transfer, resulting in the temporary disappearance of the PFBT fluorescence. After NIR laser irradiation, the PFBT was released from HGNs, resulting in fluorescence restoration. In addition, the release of RAPA was both observed *in vivo* or *in vitro* studies, thereby inhibiting the proliferation of tumor cells and promoting their apoptosis.

In addition to PFBT, other fluorescent agents were also presented for fluorescence imaging-guided cancer therapy. For example, Zhang *et al.* [164] developed a HGNs-based nanosystem with signal DNA as an imaging agent. After laser irradiation, the HGNs could be heated locally, and then the signal DNA was activated and released from double-stranded DNA, significantly providing fluorescence imaging for this nanosystem. Subsequently, the fluorescent microdiamonds (FMDs) were presented as potential imaging agents for PTT [165]. The surface of HGNs was modified with functionalized FMDs. Guided by fluorescence imaging, the nanocomposites showed uniform and local heating effects in the tumor areas, which illustrated the promising application of HGNs-based nanoplatform for PTT.

In order to monitor the deep orthotopic tumor cells, Shen *et al.* [79] have developed a new HGNs-based fluorescent biosensor. This HGNs-based nanosystem not only showed the advantage of the remarkable loading capacity but also realized the controlled release. Their results demonstrated that the fluorescence imaging strategy could detect intracellular mRNA with high specificity and sensitivity *via* separating by targeting mRNA.

### HGNs-based nanoplatform for other imaging-guided tumor therapy

In addition to the ultrasound/Raman/fluorescence image-guided tumor therapy based on HGNs mentioned above, other imaging modes have also been developed to further optimize design strategies.

For example, Bai *et al.* [166] constructed HGNs@Fe_3_O_4_-FA nanoparticles based on HGNs. They modified sulfhydryl groups on the surface of Fe_3_O_4_ and then covalently bound them to HGNs for magnetic resonance imaging/photoacoustic imaging (MRI/PA) guided PTT. Combining MRI/PA, the HGNs@Fe_3_O_4_-FA exhibited precise targeting of tumor cells and effectively promoted cell apoptosis.

**Figure 6.**
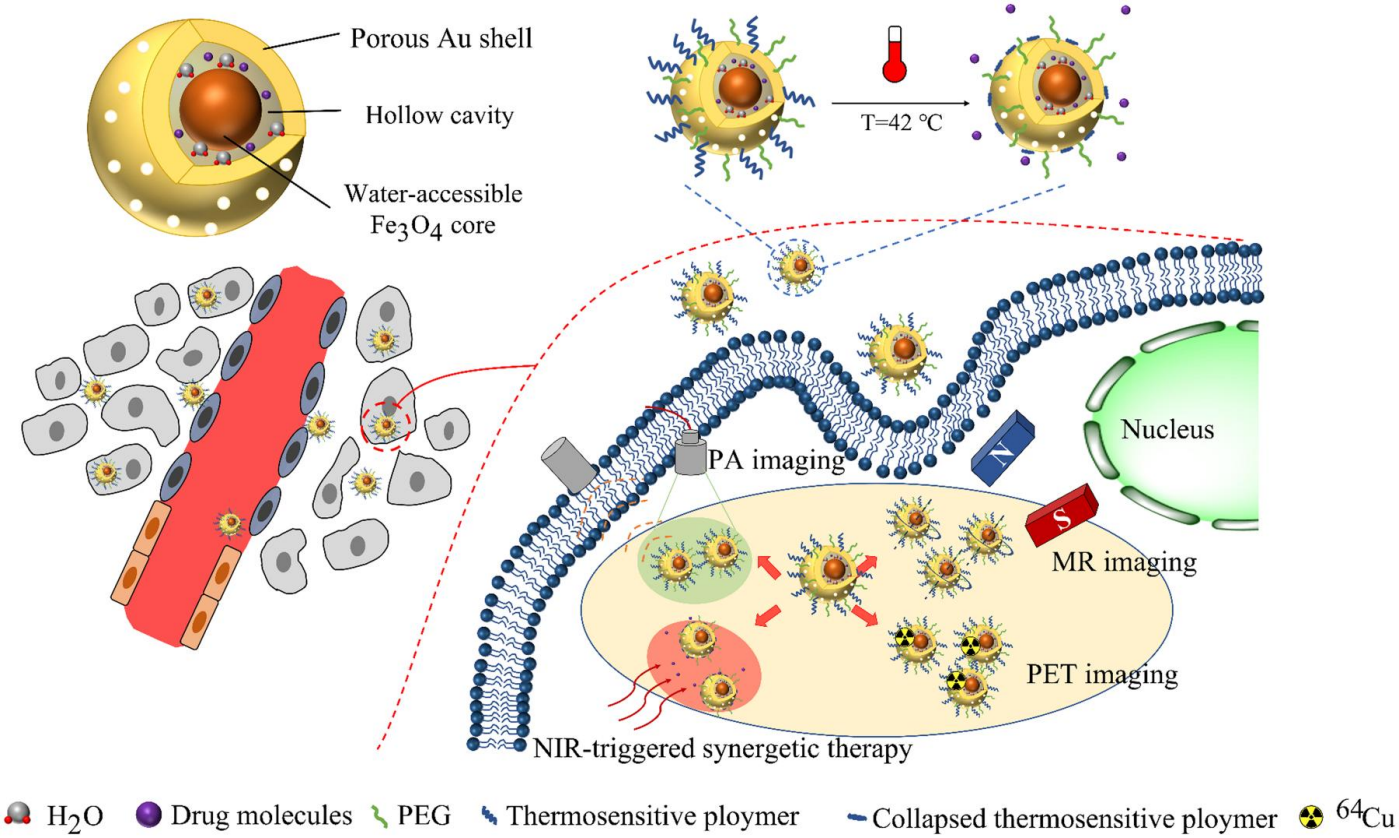
Yolk-shell Fe_3_O_4_@Au was synthesized by reverse microemulsion method with silica nanoparticles as templates. This yolk-shell Fe_3_O_4_@Au not only played a role of multimodal tumor diagnosis guided with MRI/PA/PET but also promoted the release of chemotherapeutic drugs after NIR irradiation, which realized PTT/PDT synergistic therapy [167].

The Fe_3_O_4_ nanoparticles could also be introduced as yolk-shell Fe_3_O_4_@Au [153]. Lin *et al.* prepared the yolk-shell Fe_3_O_4_@Au based on HGNs and superparamagnetic Fe_3_O_4_ nanoparticles, modified with thermosensitive poly(N-isopropylacrylamide-co-acrylamide) (whose low critical solution temperature is 40°C) (Figure 6). They first synthesized Fe_3_O_4_ nanoparticles with oleic acid as a stabilizer. The SiO_2_ obtained by the hydrolysis of tetraethyl orthosilicate (TEOS) was directly coated on the surface of Fe_3_O_4_ nanoparticles using the reverse microemulsion method, and the hydrolyzed TEOS could replace the oleic acid to coat on the surface of Fe_3_O_4_ nanoparticles. Thus, the phase transfer problem of hydrophobic Fe_3_O_4_ nanoparticles was avoided, which also effectively stabilized the Fe_3_O_4_ core and even enhanced MRI compared to core-shell Fe_3_O_4_@Au. Then, the Au shell was formed using SiO_2_ as a sacrificial template. Subsequently, in order to explore whether the yolk-shell structure can effectively mitigate the reduction in contrast performance of the MRI agents, the authors compared the T_2_ delay effect of the yolk-shell Fe_3_O_4_@Au with that of the core-shell structure. It was found that the *r_2_* value of yolk-shell Fe_3_O_4_@Au was 149.4 mM^−1^s^−1^, which was approximately 2.4 times higher than that of core-shell Fe_3_O_4_@Au (61.9 mM^−1^s^−1^). This demonstrated that the yolk-shell Fe_3_O_4_@Au did achieve the effect of stabilizing Fe_3_O_4_ core and enhancing MRI compared to the core-shell Fe_3_O_4_@Au. Moreover, they also employed a chelating agent-free ^64^Cu radiolabeling strategy to deposit ^64^Cu on the surface of HGNs directly, thus enabling positron emission tomography (PET). According to the images of MRI and PA, the signal intensity of yolk-shell Fe_3_O_4_@Au was significantly enhanced compared with that of core-shell Fe_3_O_4_@Au. In addition, after NIR irradiation, yolk-shell Fe_3_O_4_@Au loaded with DOX also showed excellent drug release and antitumor effects.

The positron emission tomography/computed tomography (PET/CT) imaging was evaluated for observing the internalization of HGNs-based nanoplatform in the tumor cells as well. The HGNs-based nanoparticles exhibited excellent internalization into tumor cells. Additionally, the ablation technique combined with embolization proved to be a feasible strategy for cancer therapy in this study [168].

## Summary and prospect

Overall, researchers possess the capability to fabricate HGNs with different sizes, shell thicknesses, surface roughness and even SPR utilizing the aforementioned techniques. It is noteworthy, however, that PTT induced by HGNs under NIR light irradiation may result in uneven heating within the tumor region, thereby rendering it incapable of eliminating tumor cells comprehensively. Consequently, the integration of PTT using HGNs with other therapeutic modalities becomes imperative. Among the diverse HGN-based therapies HGNs introduced above (Table 2), the hollow structures of HGNs provide great potential for drug delivery. Specifically, the precise targeting of drugs is achieved through the attachment of target ligands onto HGNs *via* Au-S bonds, significantly enhancing the therapeutic efficacy and optimizing the combined therapeutic approach.

**Table 2.** Summary of HGNs-based nanocomposites in cancer therapy

Therapeutic method	Nanocarrier	Size (nm)	Zeta potential (mV)	Encapsulation efficiency (%)	Minimum cell viability (%)	Type of cancer	Image mode	Ref.
Photothermal therapy	MSCs+HGNs	40.5 ± 7 (HGNs)	–	–	–	Cervical cancer and glial carcinoma	–	[101]
	HGNs-FA	–	–	–	29 ± 4.7 (Hela, NIR irradiation, 39 mW)	Cervical cancer	Photoluminescence	[102]
	HGN-AS1411	63.4 ± 4.8	−27.2 ± 0.7	–	<20 (A375, *C*_Au_ = 150 μM, 808 nm, 2 W/cm^2^, 2 min)	Melanoma	Fluorescence imaging/NIR imaging	[100]
Photothermal immunotherapy	PLGA (loading APP and HGNs)	400–800	–	37.4–65.9 (APP)	∼60 (4T1 and CT26, *C*_Au_ = 2 mg/ml, NIR irradiation, 2 W/cm^2^, 3 min)	Breast cancer and colon cancer	NIR imaging	[124]
	FAL-HGNs	151 ± 4.6	–	49.3 ± 6.1 (ICG)	∼40 (CT26, *C*_Au_ = 50 μg/ml, NIR irradiation, 1 W/cm^2^, 3 min)	Colon cancer	Fluorescence imaging	[103]
Chemo/photothermal therapy	HGNs	∼60	14.6 ± 0.5	57.3 (DOX)	<20 (SKOV3, *C*_Au_ = 5 μg/ml, NIR irradiation, 2 W/cm^2^, 3 min)	Ovarian cancer	Fluorescence imaging/NIR imaging	[130]
	Liposomes (loading DOX and HGNs)	154.7 ± 2.9	−59.2 ± 1.9	93.57 (DOX)	–	Ovarian cancer and lung adenocarcinoma	Fluorescence imaging	[131]
	PC_10_A/DOX/HGNs	37.65 ± 4.23 (HGNs)	−22.2	–	–	Liver cancer	Fluorescence imaging	[104]
	Micelles (loading PTX and HGNs)	68.6 ± 5.4	32.1 ± 1.3	>80 (HGNs and PTX)	–	Colon cancer	Ultrasound imaging/Fluorescence imaging	[134]
	FA-HGNs	46 ± 4.8 (HGNs)	–	77.98 (DTX)	<20 (PC-3, *C*_DTX_ = 116 nM, NIR irradiation, 4.12 W/cm^2^, 5 min)	Prostate cancer	SPECT imaging	[135]
	HGNs	85.6 ± 2.2	−15.1 ± 2.8	86.7 ± 3.1 (cisplatin)	16 (Hela, 808 nm, 2 W/cm^2^, 3 min)	Cervical cancer	–	[132]
	Liposome (loading HGNs and DOX)	196.6 ± 17.6	−24.8 ± 0.36 (water)/−18.6 ± 0.21 (serum)	49.27 ± 1.60 (DOX)	∼10 (MCF-7, *C*_Au_ = 10 μM, 808 nm, 1 W/cm^2^, 3 min)	Breast cancer	Ultrasound imaging/Fluorescence imaging	[154]
	HGNs	∼50	−28.4	–	13 (MDA-MB-231, *C*_cisplatin_ = 16 μM, 808 nm, 1 W/cm^2^, 5 min)	Breast cancer	Ultrasound imaging/NIR imaging	[155]
	HGNs	∼83	–	–	<20 (U87MG, *C*_DOX_ = 5 μg/ml, 808 nm, 0.5 W/cm^2^, 5 min)	Glial carcinoma	MR/PA/PET	[167]
Photothermal photodynamic combination therapy	HGNs	∼40	−16.4 (HGNs)	∼25 (Ce6)	30 (Hela, C_Ce6_=20 μM, 670 nm, 5 min)	Cervical cancer	Fluorescence imaging/NIR imaging	[140]
	HGNs	122.5 ± 13.5	−4.58 ± 0.78	35.8 (ICG)	11.6 (SKOV3, *C*_Au_ = 50 μg/ml, NIR irradiation, 1 W/cm^2^, 2 min)	Colon cancer	Fluorescence imaging	[147]
	HGNs	∼50	–	∼35.6 (ICG)	<20 (CT26, *C*_Au_ = 100 μg/ml, NIR irradiation, 1 W/cm^2^, 2 min)	Colon cancer	Photoacoustic imaging	[148]
Chemo/photothermal/photodynamic therapy	HGNs	∼55 (HGNs)	–	58 (drug loading of DOX)	∼20 (Hela, *C*_DOX_ = 79 μg/ml, 650 nm)	Liver cancer	–	[149]
Photothermal/antiangiogenesis therapy	HGCNs	95.7	−16.5	33.5 (RAPA)	19.02 (4T1, C_Au_ = 79 μg/ml, 670 nm)	Breast cancer	NIR fluorescence imaging	[105]

HGNs have also garnered significant attention in tumor diagnosis and treatment. Among metal nanomaterials, gold nanoparticles show exceptional biocompatibility, surface modifiability and low biotoxicity. In comparison to solid gold nanospheres, HGNs exhibit a more regular spherical morphology, a larger specific surface area and an improved NIR-SPR effect, which enhances surface Raman scattering and extended half-life *in vivo*. Firstly, HGNs have been demonstrated for PTT. HGNs can absorb visible or NIR light and convert the light energy to heat energy *via* SPR, while highly tunable SPR make the HGNs an ideal nanomaterial in PTA. However, PTT induced by HGNs under NIR light irradiation may result in uneven heating within the tumor region. Secondly, HGNs have been designed as nanocarriers for cancer therapy and diagnosis as well *via* the unique hollow structure. Various theranostic agents or imaging agents are covalently modified onto the surface of HGNs with Au–S bonds or non-covalently loaded within HGNs to construct a nanoplatform for cancer therapy and diagnosis. Specifically, the precise targeting of drugs is achieved through the attachment of target ligands onto HGNs *via* Au–S bonds, significantly enhancing the therapeutic efficacy and optimizing the combined therapeutic approach. Thirdly, HGNs have shown the capacity of photothermal and photoacoustic imaging. Under the irradiation of NIR, HGNs can be used for thermal imaging *via* SPR. The strong NIR absorption property of HGNs makes them wonderful contrast agents for photoacoustic imaging agents, due to the minimal absorption of human tissues and water in the range of NIR. However, both imaging strategies only observed a depth of centimeters, which remain difficult for the diagnosis of deep tumors. Finally, HGNs have been utilized as photothermal coupling agents to load other organic fluorescence agents for real-time imaging in the tumor tissue. When photosensitizers are combined with HGNs, they can temporarily be quenched. Photosensitizers exhibit non-fluorescence and decline their light toxicity *in vivo*, especially in non-tumor areas. Under the irradiation of NIR, the photosensitizers can be released from HGNs and dequench for fluorescence imaging and tumor treatment. Nevertheless, there are several barriers to clinical translation. (i) Limited penetration depth of NIR light. X-ray and down-conversion nanoparticles can improve the penetration depth of light [169]. (ii) PTT has great damage to the skin [102]. Preparing nanoparticles for mild hyperthermia may alleviate this concern. (iii) Long-term safety of HGNs remains to be investigated. In general, HGNs-based multifunctional nanoparticles show high therapeutic efficiency and excellent inhibition in tumor growth through multimodal therapy and have broad application prospects in the biomedical field.
